# The chemical succession in anoxic lake waters as source of molecular diversity of organic matter

**DOI:** 10.1038/s41598-024-54387-0

**Published:** 2024-02-15

**Authors:** Maximilian P. Lau, Ryan H. S. Hutchins, Suzanne E. Tank, Paul A. del Giorgio

**Affiliations:** 1 Interdisciplinary Environmental Research Centre, Technische Universität Bergakademie Freiberg, Brennhausgasse 14, 09599 Freiberg, Germany; 2 Département des Sciences Biologiques, Université du Québec à Montréal (UQAM), 141 Avenue du Président-Kennedy, Montreal, QC H2X 1Y4 Canada; 3 Department of Biological Sciences, University of Alberta, Edmonton, AB T6G 2R3 Canada; 4 Department of Chemistry and Biology, Toronto Metropolitan University, Toronto, ON M5B 2K3 Canada

**Keywords:** Lake-water quality, Anoxia, Carbon cycling, Chemical diversity, Carbon cycle, Limnology

## Abstract

The aquatic networks that connect soils with oceans receive each year 5.1 Pg of terrestrial carbon to transport, bury and process. Stagnant sections of aquatic networks often become anoxic. Mineral surfaces attract specific components of organic carbon, which are released under anoxic conditions to the pool of dissolved organic matter (DOM). The impact of the anoxic release on DOM molecular composition and reactivity in inland waters is unknown. Here, we report concurrent release of iron and DOM in anoxic bottom waters of northern lakes, removing DOM from the protection of iron oxides and remobilizing previously buried carbon to the water column. The deprotected DOM appears to be highly reactive, terrestrially derived and molecularly distinct, generating an ambient DOM pool that relieves energetic constraints that are often assumed to limit carbon turnover in anoxic waters. The Fe-to-C stoichiometry during anoxic mobilization differs from that after oxic precipitation, suggesting that up to 21% of buried OM escapes a lake-internal release-precipitation cycle, and can instead be exported downstream. Although anoxic habitats are transient and comprise relatively small volumes of water on the landscape scale, our results show that they may play a major role in structuring the reactivity and molecular composition of DOM transiting through aquatic networks and reaching the oceans.

## Introduction

Historically, the role of aquatic systems as C sources, sinks, pipes or reactors was attributed based on their ambient potential for organic matter (OM) burial, transport and mineralization to carbon dioxide in the circulating water column^1–3^. The last two decades have seen a surge of interest in OM quality and composition^4–6^, with many studies offering insight into the molecular footprint of dissolved OM (DOM) and how this footprint is shaped and modified during transit from land to the oceans within aquatic networks^7–9^. In this regard, DOM composition is moderated by patterns of removal or persistence, that generate ambient DOM lacking fractions that are either reactive^10–12^ or immobilized and protected^13–16^. Along the downstream network, the dynamics in molecular-level DOM complexity affect the composition of its microbial degrader community^17^, and shape their activity patterns^18^.

Oxygen-free habitats within aquatic systems are often temporarily transient and spatially constrained, and they were typically perceived as sites of modest DOM processing and mineralization^19,20^. Studies specifically targeting anoxic waters, however, have challenged this view by demonstrating relatively high rates of microbial activity^21,22^ and DOM removal^23,24^ relative to oxic environments. Moreover, the decreased stability of DOM-protecting mineral phases (notably Fe(oxyhydr)oxides) may supply anoxic waters with potentially large amounts of additional DOM^25–30^. It is currently unknown, however, how such DOM supplementation to anoxic waters interacts with the degradation patterns within these habitats, notably the restriction of anaerobic DOM mineralization to high-energy sub-pools of DOM^31,32^. Because mixing and re-oxygenation of these waters precipitates a specific fraction of the ambient DOM with metal hydroxide surfaces^33–35^, the question emerges as to what extent these processes are transient and fully reversible, or if they permanently alter DOM composition, and therefore affect carbon cycling in the interconnected surface network, potentially at the landscape-scale.

Here, we hypothesize that there are systematic shifts in amounts and molecular composition of DOM, and its subsequent fate during a seasonal cycle of lake anoxia, where we followed the DOM pool before, during and after exposure to anoxia. We have carried out our research in two northern lakes that stratify seasonally to form well-mixed epilimnia and stagnant hypolimnia, as those lakes are numerous in high-latitude regions^36^, represent 24% of the global C burial^37^, and 25% of global terrestrial surface water^38^. We used ultrahigh-resolution mass spectrometry to monitor DOM molecular composition, and compared fractions that were added, degraded or conserved when transitioning from oxic to anoxic conditions. In addition, we followed hypolimnetic Fe concentrations during the oxic-anoxic cycle because we hypothesize that DOM-Fe co-precipitation and sediment release of Fe-bound DOM could potentially be one of the mechanisms shaping DOM dynamics during the seasonal cycle. The results potentially imply a hitherto unrecognized landscape function of anoxic aquatic habitats, acting as unique reactors of mineral-protected, terrestrially derived OM from the watershed. Whereas ecosystem deoxygenation is widespread already today^39^, many current global trends—warming, eutrophication, river damming—favor its global proliferation^40–42^, with major implications to OM cycling and burial in inland waters.

## Results

**Figure 1:**
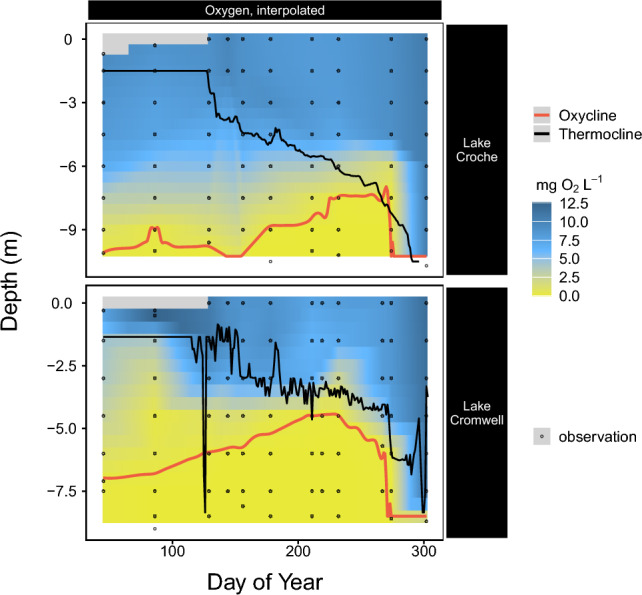
Spatiotemporal oxygen distribution in the two sampled north temperate lakes, Lake Croche (above), and Lake Cromwell (below). Data is acquired using 8–10 continuously measuring probes per lake and monthly profiles (grey dots indicate sampling positions). The black line separates the well-mixed epilimnion from the stagnant hypolimnion. The red line demarks the oxygen-free waters of the hypolimnion (threshold: 0.3 mg L^−1^, 8 µM).

**Figure 2:**
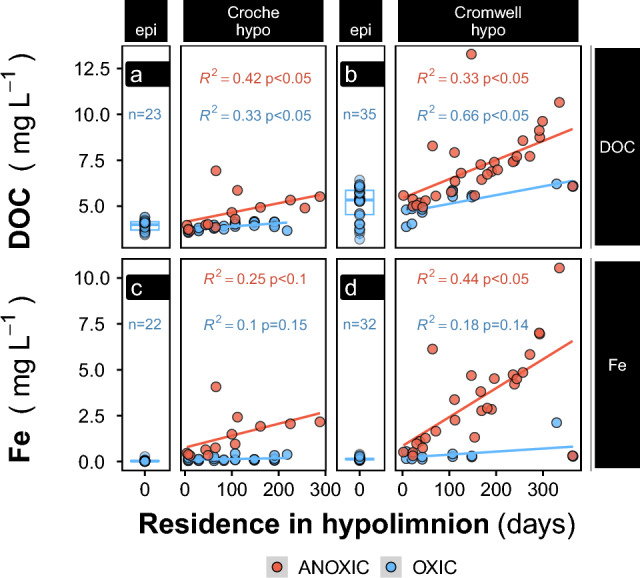
Concentrations of dissolved organic carbon (DOC, upper row), and iron (Fe, lower row) in the north temperate lakes Croche (left) and Cromwell (right). Panels named “epi” show samples taken monthly in epilimnetic (mixed surface) waters individually and summarized as box-plots (background, representing interquartile range and median). Panels named “hypo” show samples taken from the hypolimnion. Colors indicate uninterrupted redox condition during residence in either water layer. Lines show linear models for the temporal development in the oxic or anoxic portion of the hypolimnion.

We present here direct determinations of changes in DOM quantity and quality as the hypolimnia of two temperate lakes shift from oxic to anoxic conditions. The Lakes Croche and Cromwell are located in close proximity within the same watershed and exhibit relatively similar seasonal patterns of water column thermal stratification and O_2_ distribution (Fig. 1) but differ in morphometry, water residence time and average DOM-C (DOC) concentration (Table 1, “Methods” section). Whereas Lake Cromwell had persistent anoxia in the bottom waters already through spring, both lakes showed progressive expansion of anoxia to shallower water layers during summer stratification (Fig. 1). We tracked Fe and DOC concentrations as different bottom water layers crossed an oxygen threshold of 8 µM O_2_, a conservative threshold for anaerobic activity^43^, from which we derived the time each specific water layer and the contained solutes were under anoxic conditions, termed anaerobic duration^44^. The two lakes differed in the length and water column distribution of anoxia, but applying the anaerobic duration metric ensures that the patterns dependent on anoxic conditions can be compared between the two. There were systematic increases in DOC concentration in anoxic hypolimnia during stratification (Fig. 2). There was less variability in epilimnetic DOC concentrations during the same period between the lakes, which were therefore grouped (Fig. 2, boxplots). The rate of increase in DOC concentration in the anoxic hypolimnion was higher in Cromwell, where DOC concentration increased by 10 µg L^−1^ day^−1^ during the whole stratification period (Fig. 2), as opposed to Croche, where DOC increased only by 5 µg L^−1^ day^−1^ (red regression fits in Fig. 2a, b). These rates represent depth-independent averages of the anoxic water layers. In both lakes, DOC and Fe concentrations in bottom waters were tightly coupled (R^2^ of 0.62–0.97, Fig. S1b), and the rates of hypolimnetic Fe accumulation differed between Cromwell and Croche by the same proportion as for DOC, converging at a constant C:Fe ratio of around 3 mol mol^−1^ (Fig. S2).

**Table 1:** Selected properties of sampled lakes.

	Cromwell	Croche
Lake area (km^2^)	0.102	0.179
Mean depth (m)	3.5	4.6
Altitude	336	359
Water residence time (years)^a^	0.06	1.88
Surface P (µg L^−1^)^b^	10.1 ± 3.3	5.0 ± 0.9
Chlorophyll-*a* (μg L^−1^)^c^	2.5	1.4

^a^^72^, ^b^this study, 2017 (mean ± SD), ^c^data from 2015 and 2016 in^75^.

### DOM released to anoxic waters

**Figure 3:**
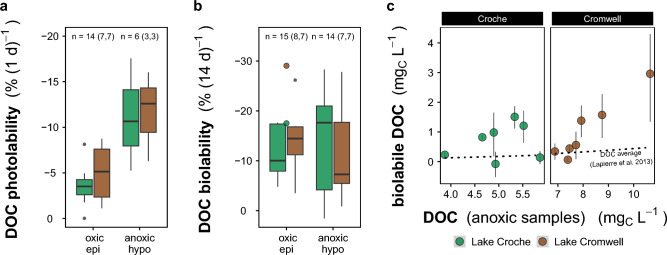
Susceptibility of DOM-C (DOC) pools to biological and photochemical processing for each lake. (**a**) DOC photolability as determined from DOC mineralization during 24 h of light exposure in a photoreactor. Considering diel light cycles and the natural light intensity in northern regions, this would roughly correspond to 6 days of natural light exposure. Values from assays are summarized as boxplots per lake (boxes indicate interquartile range, line shows the median, black points indicate samples away from the interquartile range by more than 1.5-fold). (**b**) DOC biolability measured in bioassays that retain the ambient redox condition [epilimnion: oxic (left), hypolimnion: anoxic (right)]. Colored points show samples from oxic waters after lake mixis introduced hypolimnetic DOC to the epilimnion. (**c**) Biolabile DOC in anoxic bioassays (y-axis) related to the DOC of anoxic samples from the hypolimnion (x-axis). The line indicates a broad average of labile DOC in samples from aquatic environments (models in^45^ Fig. 1a and Fig. S1).

Linked to the co-occurring release of DOC and Fe during anoxia, the photo- and biolability of DOM shifted. Both color (cDOM, Figs. S1a and S3) and photolability (Fig. 3a) of the DOM were higher in anoxic waters than in oxic surface waters. The biolabile portion of the DOM pool, as measured in dark bioassays that retained the ambient redox conditions, was low shortly after oxygen depletion, but increased with increasing DOM released into the hypolimnion (Fig. 3c, Table S1). When pooled, this dynamic results in a large variability of biolability in samples from the anoxic hypolimnia (Fig. 3b). In surface waters, biolability did not differ from the pooled samples of the anoxic hypolimnion (Fig. 3b) and was potentially influenced by drivers operating in oxygenated epilimnetic waters that were not under investigation here, e.g. delivery of fresh terrestrial DOM in response to precipitation or aquatic-terrestrial coupling, algal production or shifts in photo-processing^11,45^.

### Molecular-level DOM composition changes

**Figure 4:**
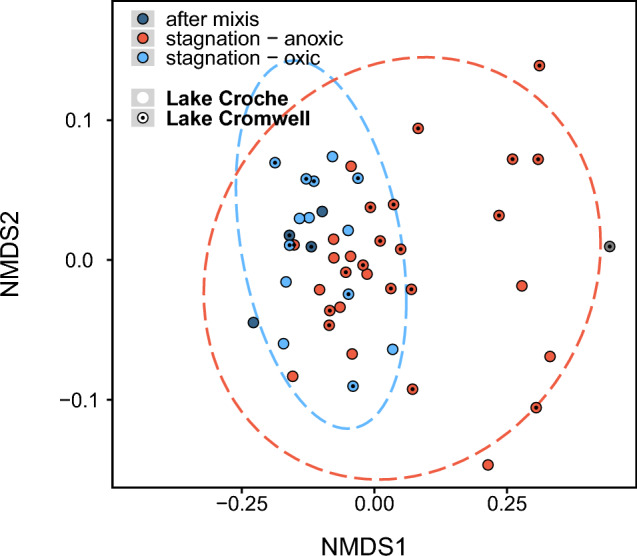
Formula presence/absence within samples used in nonlinear multidimensional scaling (NMDS) to visualize the level of chemical sample similarity. 2-dimensional stress = 0.09. Symbols indicate DOM origin, color reflect ambient oxygen availability. Ellipses delineate standard deviations around group averages of DOM from oxic and anoxic waters, respectively. One sample taken under ice was eliminated from the dataset (grey, right) because of the unknown redox history of this sample from the first campaign.

We explored the molecular-level composition of DOM in 49 samples recovered from oxic (n = 18) and anoxic (n = 31) compartments of the lakes (n = 21 for Croche) throughout the year by ultrahigh-resolution Fourier transform ion cyclotron resonance mass spectrometry (FT-ICR-MS; Methods). We identified > 7200 unique molecular formulae over a mass range of 145–920 Da and their relative abundance in each sample, and explored their presence/absence patterns through nonmetric multidimensional scaling (NMDS). NMDS ordination revealed distinct shifts in molecular composition mediated by ambient oxygen presence or absence (Fig. 4): there was a relatively close clustering of molecular DOM composition among samples from oxic waters, collected during stratification in surface and deep waters and during fall overturn. In contrast, there was a clear expansion of the variability among samples from anoxic waters, suggesting large increases in heterogeneity in the molecular structure under anoxic conditions (Fig. 4).

**Figure 5:**
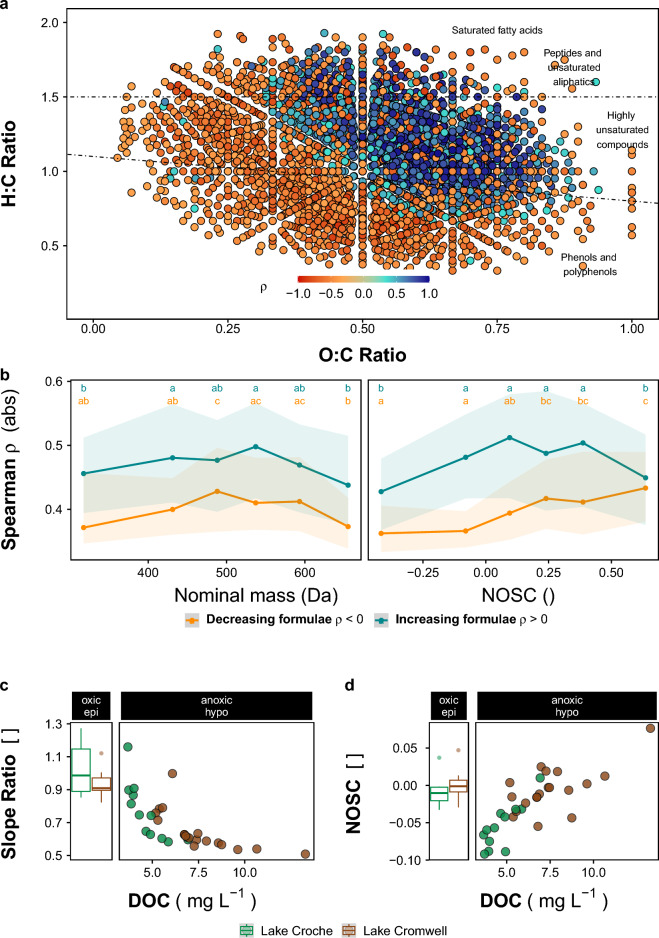
Molecular-level DOM patterns in oxic and anoxic waters of northern lakes **(a)** Color-coded correlations (ρ) of molecule-specific intensities with the anaerobic duration of samples, t_a_, (n = 49) identifies the molecular subpopulations in Van-Krevelen space that lose (orange) or gain (blue) intensity with longer t_a_. Only molecules with correlations higher or lower than ± 0.33 are shown (n = 3514). Permutation test indicates the result is highly significant (p < 0.01). Plotting order was random. **(b)** Absolute values of the spearman correlation coefficient (ρ) of formulae that belong to the losing (orange) or gaining (blue) subpopulations of DOM. Formulae were binned to 6 equally occupied bins according to their nominal mass (left) or nominal carbon oxidation state (right) to show how coupling varies with molecule properties. Lines show median, shaded areas delineate the range between the 25th and the 75th percentile (“moving boxplot”). Only coefficients of significant correlations (p < 0.0251) are used. Letters indicate similarity at the *p* = 0.05 level. **(c,d)** Bulk properties of the DOM pools. Panels named “oxic/epi” show samples taken monthly in epilimnetic (surface) waters as box-plots (representing interquartile range and median). Panels named “anoxic/hypo” show samples taken from the anoxic hypolimnion. **(c)** Spectral slope ratio, indicates larger molecular sizes when decreasing^47^. **(d)** Nominal carbon oxidation state (NOSC), increasing with DOC concentration in anoxic waters. The bulk changes in anoxic DOM pools are consistent with molecular-level assessments in **(b)**, showing that increasing high-mass and high-NOSC molecules are not only more abundant (formulae per bin) but also increase more rapidly than they are being consumed.

In order to explore the molecular-level shifts underlying the observed divergence of DOM composition during anoxia, we paired the patterns of individual formulae intensity with the duration of exposure to anoxia using canonical correlation analysis (Fig. 5a). The overall DOM molecular composition was significantly (p < 0.001) correlated to the anaerobic duration. We further identified individual molecular formulae that drove this pattern and that correlated with anoxia, by performing Spearman’s rank correlations for each formula^12^. Spearman correlation is a non-parametric measure of the (degree of a monotonic) relationship of two variables, has reduced sensitivity to outliers and no assumptions about the underlying distributions. Formulae were grouped by Spearman correlation coefficients (ρ) in order to distinguish two trends: 1) formulae with significant, positive ρ that tend to be more abundant, from 2) formulae with negative ρ that tend to be less abundant with ongoing exposure to anoxia. Van-Krevelen visualization shows distinct molecular populations associated with these different trends, with formulae of higher O:C and H:C ratios increasing during anoxia (Fig. 5a, p < 0.0251, n = 1844). Partitioning the formulae in two classes (negative and positive ρ) also allowed for the comparison of coupling strength with individual formulae characteristics (Fig. 5b)^46^. There were slightly more formulae with significant increasing than with decreasing trends (981 vs. 863 decreasing, p < 0.0251). These formulae were added to the DOM pool, consistent with the general increase in DOM pool size in anoxia, and had higher O:C values (Fig. 5a). Formulae more consistently adhering to the increasing trend had approximately zerovalent nominal oxidation state of C (NOSC) and had higher absolute correlation coefficients than formula that were decreasing (p < 0.01) (Fig. 5b). These observations were also consistent with bulk trends in DOM quality: the spectral slope ratio, indicative of average molecular weight^47^, decreased with increasing exposure to anoxia (suggesting more DOM components with higher average weight), and sample weighted average NOSC increased with increasing concentration of DOC (Fig. 5c, d).

Formulae with a negative correlation coefficient relative to anaerobic duration experienced either a conservative behavior or a net decrease during exposure, because the overall DOM pool increases along the gradient of anaerobic duration (Fig. 2). The higher the absolute value of coupling (|-ρ|), the more likely these components were degraded or removed as opposed to diluted by the addition of new DOM. Across all formulae with negative ρ, the strength of coupling increased with increasing formula NOSC (Fig. 5b).

### Is OM mobilization transient?

**Figure 6:**
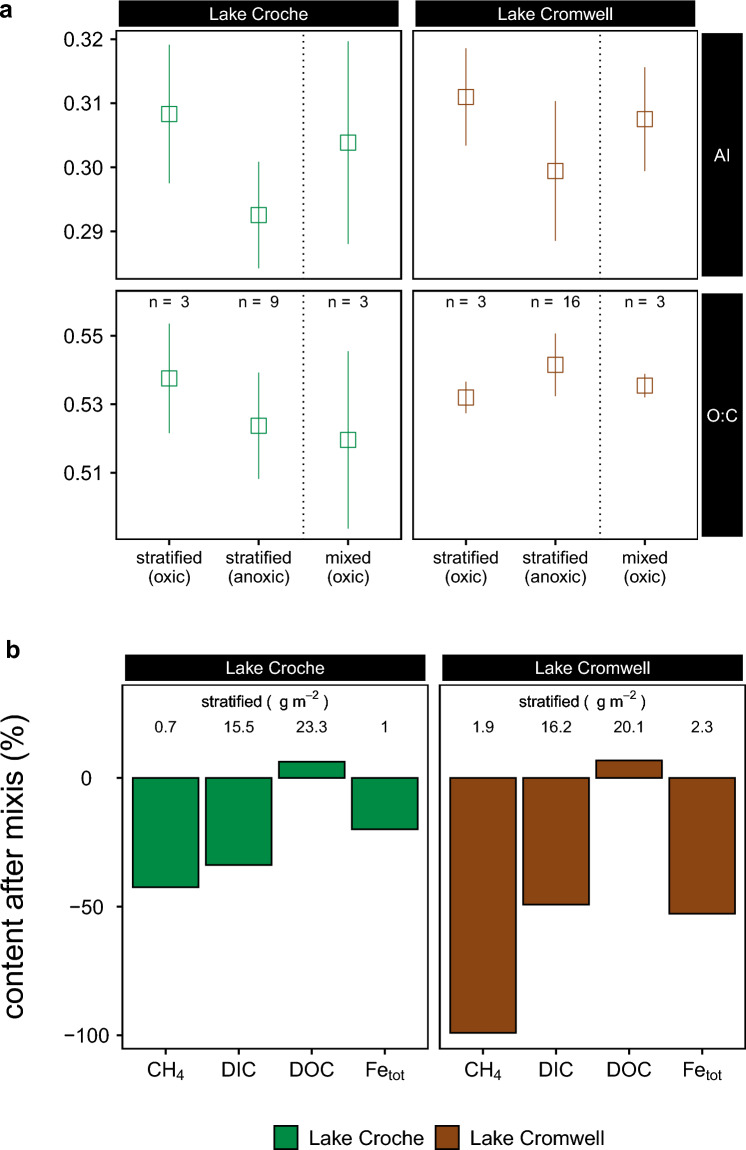
**(a)** Changes in DOM quality parameters shown as averages of samples from anoxic and oxic compartments of the water column at maximum expansion of anoxia (shortly before mixis, left) and after autumn mixing homogenized these two compartments (right). **(b)** Relative changes in the areal abundance of carbon species (CH_4_, DIC, DOC) and Fe before and after autumn circulation (mixis). Values indicate areal content along the whole water column of species during maximum expansion of anoxic waters. Bars indicate the relative elimination of the species (in %). Positive values for DOC indicate net increase in DOC during the time of autumn circulation.

The mixing of oxic and anoxic waters can trigger metal (oxy)hydroxide formation, thereby potentially precipitating parts of the bottom-water DOM^15^ and effectively reversing the previous release. Because only DOM withstanding immediate precipitation has a lasting effect on the landscape C balance, we studied the efficiency of the DOM elimination by comparing pre- and post-overturn DOM composition and water column abundance of DIC, CH_4_, DOC and Fe (Fig. 6). We found greatly reduced areal gas stocks (total CO_2_ as DIC, CH_4_) post-overturn of which a large portion likely vented to the atmosphere. Oxygenation of the water column also markedly decreased Fe concentrations, although about half of the Fe that accumulated in the anoxic hypolimnion remained in the water column, at least for a period of several weeks. DOC, in contrast, was relatively unaffected by the overturn event, during which the areal DOC stock experienced a small net increase (Fig. 6b). The aromaticity index, a quality metric derived from DOM molecular composition^48^, showed a clear mixing pattern for DOM with significantly greater values in oxic than anoxic waters prior to mixing, and average values thereafter (Fig. 6a). The elimination of Fe without effective co-precipitation of DOM suggests that DOM released from mineral protection and processed under anoxic conditions, is left to mix with the surface waters and can undergo further processing within the lake and transport and processing in the downstream aquatic network.

## Discussion

We have shown that lake stratification and the onset of anoxia triggers the addition of significant amounts of DOM to hypolimnetic waters. We interpret this as release of DOM from sediments together with Fe (Fig. 2), similar to reports from other lacustrine systems^25–27^. Such coupled release suggests that Fe plays a major role in moderating the sink and source behavior of DOM at sediment surfaces, similar to the Fe-driven redox cycling of PO_4_^3-^, in which a small layer of Fe may be responsible for pronounced release and capture of a surface-bound solute^49^. Fluctuating redox conditions at the sediment–water interface leads to natural Fe accumulation in sediment surfaces even when waters are low in Fe^50^.

Of the organic carbon that reaches a lake, a fraction accumulates and remains stored in sediments for long timespans^1^. Among the various controls on the storage function^37,51^, oxygen availability may be the most critical, as sediments with a lower oxygen exposure time (OET) were found to bury more and respire less OM^20^, as indicated by a higher organic carbon burial efficiency (OCBE, the ratio of burial to deposition, in %). Clearly, when large amounts of DOM are released from sediments, as shown here for lakes with anoxic hypolimnia, the OCBE is affected. Although release of DOM from sediments in anoxic waters has been widely reported^23,52,53^, this mechanism has rarely been considered when estimating OCBE. In boreal lakes, Peter et al.^53^ observed that anoxic lake sediments release DOC equivalent to 9–40% of the C they bury, but incubations suggested effective re-precipitation after return to oxic conditions, so that the overall impact on OECB was considered low. In our work, however, we find that the strong release of DOM was not accompanied by a rapid removal by mineralization or precipitation, suggesting that DOM release may, where active, invisibly yet strongly reduce OCBE when calculated commonly as the balance between deposition and burial (measured by sediment traps and dating, respectively). In lakes Croche and Cromwell, the measured DOM release (Fig. 2a, b) translates to areal rates of 0.5 and 1.2 g m^−2^ year^−1^ (see SI). Estimates for the deposition rates derived from a sinking flux-Chl.a relationship^54^ of 30 and 43 g_C_ m^−2^ year^−1^ (Croche, Cromwell) are similar to observations in lakes of similar location and biogeochemistry, varying between 10 and 50 g_C_ m^−2^ year^−1^^55^. The latter study also showed that the estimates for OCBE are strongly correlated with the lake size (p = 0.003, R^2^ = 0.62). Applying this relationship for the lakes studied herein, anoxic DOM release at the observed rate would suggest that 1–7% (Lake Croche) or 4–21% (Lake Cromwell) of deposited OM would be released as DOM, instead of mineralized to CO_2_. If, however, OCBE was calculated using burial and mineralization rates (as e.g., in^20,56^), this DOM release would be unaccounted for, rendering OCBE seemingly higher. Interestingly, it is anoxic sediments, where DOM release would be most pronounced and that are classically considered to have the highest OCBE^20^, where this overestimation would be greatest. A potential for OCBE overestimation is supported by a unique case study where OCBE was calculated independently with rates of burial and either deposition or mineralization, showing an OCBE mismatch of 6–13%^57^. Although this difference may seem minor, it should be noted that the imbalance of deposition and release in lakes is the only active mechanism in freshwater networks that removes carbon from atmospheric circulation^58^. Because its magnitude is considered significant on the global scale^2^, uncertain or unaccounted mechanisms may critically exacerbate efforts of constraining the role of lakes in the global carbon cycle.

Lake sediments receive both autochthonous and allochthonous detritus (i.e., algal or terrestrial OM, respectively). Teodoru et al*.*^59^ found the particulate OM settling in northern lakes of Quebec to be on average 65% terrestrial (range: 20–100%). Additionally, DOM has been shown to be an important precursor for the formation of particles, and their flocculation is a major vector of OM to sediments in high-latitude ecosystems, thereby imparting a strong terrestrial signature^60–62^. Our results suggest that coprecipitation of OM with Fe is likely another major vector of C to lake sediments. Because Fe and DOM are ubiquitous components of aquatic networks, Fe particles could have been loaded with freshly leached DOM early on, presumably directly after their first flocculation (under oxic conditions), for example at the soil-stream-interface^33^. The molecular characteristics of the DOM that accumulates in anoxic water matches the high O:C values of DOM with a high tendency for Fe sorption^33,35^. The redox-dependent, physical protection by Fe^14^ inhibits the participation of these Fe-associated DOM pools in the usual transformation/mineralization cascade of DOM in the aquatic continuum^10,11^. This DOM therefore represents a specific pool that can be considered fresh and both biologically and photochemically reactive to an extremely high degree: biolability assays showed the anoxic hypolimnion continuously acquired labile C and reached proportions to total DOC that are much higher than what is found both in the epilimnia (Fig. 2b) and typically in surface waters (mostly < 10% in a large-scale survey^45^). Further, the mostly unaltered terrestrial character of the anoxia-released DOM is confirmed by its optical properties: this DOM was intensely colored (Figs. S1a and S3) and highly photo-labile (Fig. 3a), even by the standard of terrestrial-derived DOM (lake and wetland cDOM median 2.52 and 6.45 m^−1^, respectively^45^). This pattern of consistent increase in DOM biolability with length of anoxia supports our hypothesis that anoxic conditions unlock previously protected portions of DOM, and that this newly released DOM is vulnerable to microbial processing, and is also highly photoreactive. All the evidence point to fresh, highly reactive terrestrially-derived DOM being released from the sediment during seasonal anoxia, suggesting a Fe-mediated shunt of this material from the watershed to the bottom of lakes.

**Figure 7:**
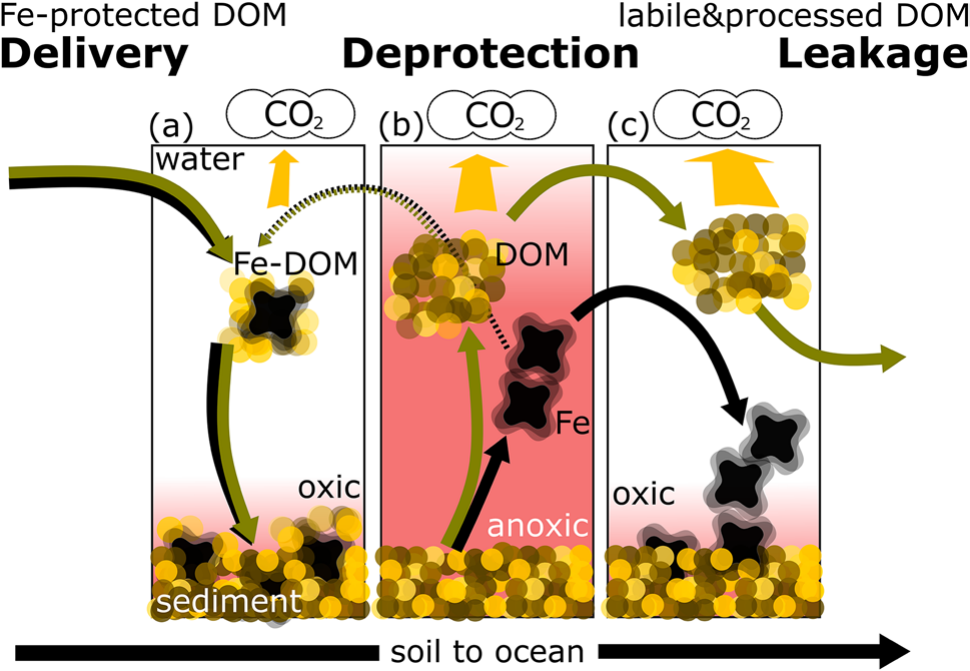
The leaking carbon cycle between hypolimnion and sediment, driven by redox fluctuations (oxic/anoxic) of the water column. Labile organic matter (yellow) is delivered to lakes in protected form on particulate but mobile Fe oxides (black). Anoxic conditions break up this protection so that labile terrestrial carbon is released to the water column. Reoxidation of the hypolimnion does not precipitate the larger share of the DOM pool, which instead may be mineralized or transported downstream.

Our results on coupled DOM and Fe dynamics and the associated DOM composition shifts therefore suggest a scheme wherein (1) terrestrial DOM associated with Fe (Fig. 2 and Fig. S2), and also (2) DOM generated continuously during early diagenesis of sediment OM (2) are both released when unaffected by adsorption to Fe(oxy)hydroxides (Fig. 7a, b). The onset of anoxia in the overlying hypolimnion solubilizes Fe and erodes its temporary storage function, but once oxygen re-precipitates Fe, it settles without a DOM co-precipitate (Fig. 6b). The high areal Fe load (Fig. 6b) suggest legacy Fe to dominate the release-precipitation cycle, with the consequence that Fe re-loads DOM post-depositionally^63^ (Fig. 7c). Such a leaky DOM-Fe cycle that releases DOM bursts from sediment to the water is amplified by the ambient pH dynamics: low pH during anoxic conditions due to high ambient CO_2_ concentrations promotes Fe dissolution from the sediment, whereas CO_2_ venting during mixing and reoxygenation increases pH and thereby Fe precipitation and OM solubilization. This cycle of Fe-associated DOM mobilization and re-precipitation implies on the one hand that a portion of the mineralization of settled OM actually occurs in the water column during periods of pelagic anoxia, when this OM is not under the protection of Fe (Fig. 7b). As such, the anoxic hypolimnetic water column is acting as an extension of the sediment diagenesis process. On the other hand, there is evidence of the cycle’s leakiness beyond the lake boundary, because a portion of the DOM that is remobilized from the sediment and reworked in the anoxic water column escapes re-precipitation, is incorporated into the lake DOM pool and eventually transported and processed elsewhere^30^, different from the mostly lake-sedentary Fe^64^.

To date, little is known about the molecular-level characteristics and dynamics of anoxia-released DOM. Anoxic conditions are known to limit microbial processing of particulate OM^20,65^, however, it is much less clear if such rate limitation also affects DOM^52^. In fact, microbial turnover was reported to be higher in anoxic than in oxic waters, and because DOM consumption is classically observed in oxic hypolimnetic waters^66^, equally or more vigorous processing may occur in anoxic hypolimnia. As a result, the composition of the ambient DOM pool may be driven not only by addition of fresh DOM, but also by its mineralization and microbial reworking. In this work, we used ultrahigh-resolution mass spectrometry to individually analyze these drivers and found that the release of fresh, terrestrial DOM during anoxia dominates the molecular imprint of ambient DOM, for example by increasing its O:C ratio and molecular weight. DOM with high O:C ratios and oxidation state was also released from soils when anoxically incubated^67^. At the same time, patterns in the anoxia-consumed sub-pools of DOM (Fig. 5b) are consistent with reported fractionation of the DOM pool when used as substrate for anaerobic respiration: lower energy yields limit microorganisms to the consumption of high-energy (high NOSC) sub-pools of the DOM^24,31^. Such DOM fractionation through preferential use of high-NOSC OC was confirmed for anoxic soils and sediments^24,32^, and dissolved carbon in ground- and sediment porewater^23,68^, where mineralization slowed when these OM fractions were depleted. For the first time, we herein show a similar DOM consumption pattern along the studied natural anoxia gradient in a fully pelagic environment. Because we simultaneously analyzed the patterns in both increasing and decreasing DOM components during anoxic conditions, we find two independent trends for the high-energy sub-pools of DOM: their preferential degradation, but also their continuous supplementation through sediment DOM release. As a result, our data suggest that the DOM that is added to hypolimnetic waters acts to release the energetic constraint on microbial metabolism during anoxia. A similar scheme was reported for an aquifer system in which extremely dilute high-energy DOM released an unreactive (i.e. low-energy) yet large pool of DOM from mineralization constraints upon mixing^68^. Both scenarios may pose as examples for so-called “activated control points”, considered to be mostly hidden from view, but critically important for the understanding of ecosystem-scale functioning^69^.

DOM has been shown to be highly diverse across inland waters^9,12^ yet the underlying drivers are still unconstrained. We observed a diversification of the molecular footprint of the DOM under anoxic condition relative to the DOM from surface waters (Fig. 4), which we attribute to processing by anaerobes^23^, and partly to reaction with their oxygen-sensitive metabolites^70,71^. DOM molecular diversity itself has been hypothesized to be a critical control on its degradation, where the dilution of individual molecules presents the final limitation for microbial uptake and mineralization^18,68^. Our results suggest that Fe-mediated re-introduction of highly reactive, terrestrially derived DOM from lake sediments, uniquely processed in anoxic lake hypolimnia, and subsequently incorporated to the lake DOM pool and transported downstream, represents a process generating DOM molecular diversity in northern inland water networks. Although these anoxic habitats are transient and may comprise a relatively small portion of the total volume of lakes, our results show that they are nevertheless hot spots in the restructuring of the molecular composition of DOM transiting through aquatic networks and reaching the oceans, which may promote its long-term stability in the (marine) sink.

To conclude, our results suggest an unforeseen role of aquatic ecosystem deoxygenation in the landscape: that of creating reaction sites that unlock, transform and mineralize mostly terrestrial, mineral-protected OM. This function is critical because the release of protected OM represents C that may have been considered removed from atmospheric circulation^37^. It is premature to extrapolate our results to the regional or global scale, because we still do not know how the delivery of Fe-bound terrestrial OM varies across major landscape types; however, we can safely assume that our results are not simply a localized phenomenon. There is clearly still much to be learned concerning the dynamics of carbon in anoxic landscape compartments, but our results suggest that anoxic waters must be viewed as an integral part of the landscape C decomposition system, because key reactions in the C cycle may only occur here. Our understanding of the aquatic-terrestrial C cycle and lakes as C sinks is, thus, incomplete without the inclusion of anoxic processes, particularly in water-rich northern landscapes.

## Methods

### Lakes and sampling

Lac Croche is a small, oligotrophic lake on the premises of the biological field station of the Université du Montréal (45.99° N, 74.00° W). Lac Croche has been the subject of intense limnological studies for the past two decades, and there is ample background information available, including estimates of planktonic primary production and sediment and water column respiration^72–74^. Lac Cromwell is a small, oligotrophic lake situated close to Lac Croche. Both are headwater lakes surrounded by a watershed dominated by maple (*Acer saccharum*) and yellow birch (*Betula alleghaniensis*) settled on well-drained Ferro-humic podzols.

Lakes were visited 10–12 times (Croche: 10) in 2018. During each visit, lake water was collected along vertical profiles of 1.5 m intervals at the deepest location of the lakes by a peristaltic pump system (n = 74 and 84 for Croche and Cromwell, respectively). Samples for quantification of nutrients and iron were collected without filtration. Samples for quantification of dissolved carbon species (DOC and DIC) were filtered through 0.45-μm filters (Sarstedt AG & Co, Germany) into acid- washed glass vials with plastic caps lined with Teflon and rubber septa and kept cold in the dark until analyses within days. Samples for mass spectrometric determination of chemical composition were filtered and stored frozen in polyethylene bottles until extraction. Further, pumped water was used to determine the concentration of CH_4_. These water samples were collected into two 60-ml gastight plastic syringes equipped with a two-way Luer-lock valve. A headspace was created in the syringes by adding 30-ml artificial (C free) air followed by vigorous shaking for 2 min. The equilibrated air was immediately transferred to 12-ml pre-evacuated exetainer vials (Labco Ltd., UK).

Additionally, we carried out vertical profiles (in 0.5 m intervals) of temperature, dissolved oxygen, conductivity, and pH using a multiparameter probe (Yellow Spring Instruments, USA). The O_2_ probe was calibrated with water vapor-saturated air at ambient pressure and surface water temperature before each profile measurement, and in 2-months intervals in a two-point calibration scheme using deoxygenated water.

### Laboratory analyses

DIC and DOC concentration were measured on an OI‐1010 TIC‐TOC Analyzer (OI Analytical, College Station, TX, USA) using wet persulfate oxidation and calculated as mean of 2–3 injections (standard errors ranged from 0 to 0.16 mg L^−1^ (DOC) and 0.3 mg L^−1^ (DIC), mean relative standard error was 1.0 and 1.1% (DOC, DIC), manufacturer detection limit stated as 2 µg_C_ L^−1^). Total phosphorus was analyzed spectrophotometrically after persulfate digestion. Total iron samples were stored acidified (pH < 1, HNO_3_) until measurement on an atomic absorption spectrometer using a graphite furnace (GBC-ARL Scientific 906AA, VIC, Australia). CH_4_ concentration was determined using a cavity ring down spectrometer equipped with a Small Sample Isotopic Module (SSIM, Picarro G2201-i, Picarro Inc., CA, USA). Concentrations in the water were then back-calculated using in situ temperatures, temperature during equilibration, gas solubility, and Henry's law^76^.

Absorbance of DOM (colored DOM or cDOM) was measured from 230 to 700 nm using a Ultrospec 2100 spectrometer (Biochrom, Cambridge, UK) with a 2 cm quartz cuvette. Absorbance measurements were corrected to Nanopure water and cDOM was calculated as the absorption coefficient at 440 nm (m^−1^, Naperian units) as CDOM = 2.303D∕r, where D is the measured absorbance and r is the cell pathlength in meters^77^. We also derived the spectral slope ratio S_R_^47^, a proxy of molecular weight, by calculating the ratio of natural-log transformed absorbance slopes between 275–295 and 350–400 nm.

### Lability bioassay

We carried out standardized biological degradation experiments that retained the ambient redox condition. Two subsamples from one sample cast were used as replicates for biological degradation experiments and dispensed in acid-washed glass bottles (500 mL). Bottles were filled bubble-free and closed with 0.5 cm thick bromobutyl rubber stoppers (Duran, GL45, Wertheim, Germany) for anoxic incubations. Oxic incubations were carried out in water that was filtered through 2.8 µm pore size GF/D filters (Whatman, Maidstone, UK) to exclude microbial grazers. Water samples were incubated in the dark at fixed temperature (21 ± 1 °C). DOC was determined on 4 to 5 timepoints throughout a 14-day incubation period by taking subsamples from incubation bottles. Samples were stored after acidification to pH 2 so that full sample series were analyzed in one instrument run. In anoxic conditions, samples were taken under N_2_ reflux by penetrating the rubber septa with two needles. We fitted first-order decay curves to the temporal DOC concentration decrease and report lability as fractions of the DOC pool that was degraded after 14 days according to the fitted curve. Our fixed-temperature approach aimed at removing or minimizing site-specific features such as lower local temperature and light exposure and thus to ensure that the resulting potential for DOC to be mineralized (lability d_14_) was comparable across the entire spectrum of sites and timepoints of this and other studies^45^.

### Residence in hypolimnion

Both lakes have been instrumented with autonomous sampling platforms, which provide high-frequency water column profiles of temperature (6–8 loggers per lake) and O_2_ (3–4 loggers per lake). We used temperature data and the *LakeAnalyzer* R package^78^ to derive the thermocline depth on hourly scale. We use the thermocline depth to delineate the well mixed epilimnetic surface waters from the stagnant water of the hypolimnion. We further sub-divided the hypolimnion into an oxic and anoxic compartment. We used logger data and biweekly to monthly oxygen profiles to assemble an interpolated oxygen distribution in the water column across space and time (in increments of 0.5 m and 1 day). All waters with an O_2_ concentration below the threshold concentration of 0.3 mg/L (8 µM) were considered “anoxic”. For all anoxic water samples, we estimated the time t_a_ for which a sample from a specific depth has remained uninterruptedly in anoxic conditions by

$${t}_{a}={t-t}_{{O}_{2}}$$

where t is the timepoint of sampling (day of year) and ${t}_{{O}_{2}}$ the (most recent) timepoint with O_2_ concentrations above the anoxia threshold^44^. This duration under which water is dominated by anaerobic processes, the anaerobic duration, t_a_, can be estimated under the assumption that in the stagnant hypolimnion, vertical transport is restricted to (negligible) diffusion^79^. This assumption implies that water of a specific depth only exchanges solutes with the contiguous sediments, a widely used simplification and e.g., the prerequisite to individually compute benthic and pelagic respiration rates from oxygen profiles^80,81^.

### High-field mass spectrometry

DOM was analyzed by FT‐ICR‐MS to assess its molecular composition. Prior to analysis, samples were subjected to solid‐phase extraction with a modified polymer‐type sorbent (100 mg Bond Elut PPL cartridges, Agilent Technologies) which was eluted with methanol^82^. Depending on a sample’s DOC concentration, the volume used for extraction was adjusted such that 40 µg of C was eluted with 0.7 ml of HPLC-grade methanol into a pre-combusted glass vial assuming a constant extraction efficiency of 65%^82^. Eluates were injected into the electrospray ionization (ESI) source of a 9.4 T FT‐ICR‐MS (Bruker Apex-Qe, Billerica, USA) in negative ionization mode at the University of Alberta (Edmonton, Canada) at a flowrate of 2 µL min^−1^ and collected over 300 added scans. This machine took part in a global interlaboratory comparison that illustrated cross-laboratory validity of FT-ICR-MS results^83^. The resulting masses were processed for quality (S/N > 4) and assigned molecular formulae based on published rules^8,12,84^ within the bounds of C_1−100_H_1−200_N_0−4_O_1−70_ P_0-1_S_0−2_ using ICBM-OCEAN^84^. The processing resulted in a final data set of > 7200 formulae that were unambiguously assigned. The contribution of each formula to a sample’s total composition was calculated by rescaling all formula peak intensities so that the total ion count for the entire spectrum was equal to 100%. Using rescaled peak intensities can reveal drivers of DOM quality better than using presence/absence information^12^, although there may be DOM components that are too small to be detected (and missing from the data) or that may be poorly ionized by ESI (and underrepresented in the analysis)^7^. Using the molecular formula, results can be displayed in Van-Kreleven-space, i.e., molecular ratios of O:C and H:C on coordinate axes. For each formula we calculated elemental ratios (mol mol^-1^), the nominal carbon oxidation state (NOSC) and the modified aromaticity index (AI_mod_) according to

$${\text{AI}}_{{{\text{mod}}}} = \left( {{1 } + {\text{ C}} - 0.{\text{5O}} - {\text{ S}} - 0.{\text{5H}}} \right)/\left( {{\text{C }} - \, 0.{\text{5O}} - {\text{ S}} - {\text{N }} - {\text{ P}}} \right){\text{ and}}$$

$${\text{NOSC}} = - \left( {\left( { - z + {4}a + b - {3}c - {2}d + {5}e - {2}f} \right)/a} \right) + {4}$$

For AI_mod_, half of the oxygen is considered present in carbonyl functional groups, assessing the amount of aromatic structures; from Koch and Dittmar^48^. For NOSC, *z* corresponds to the net charge of the organic compound, and the coefficients *a*, *b*, *c*, *d*, *e*, and *f* refer to the stoichiometry of C, H, N, O, P, and S. To limit NOSC uncertainties due to assumptions of average oxidation state of heteroatoms (S and P^23^), we only used CHON molecules when estimating NOSC. The NOSC of an organic compound can be used to predict Gibb’s free energy of reaction, Δ*G*_rxn_^31^ for its oxidative half-reaction, and therefore presents a simple approach to calculate the thermodynamic driving force for mineralization reactions, both under oxic and anoxic conditions^32^.

### Statistics

To illustrate trends in DOM molecular composition data over mode and duration of redox condition, we used two-dimensional non-metric multidimensional scaling (NMDS), an ordination technique to graphically represent differences between multiple populations. In this analysis, individual molecular formulae represent individual classes of compounds that were either present or absent (intensity ≥ 0) in a sample. Canonical analysis of relative intensities of all formulae was used to explore the relationship between DOM molecular composition (from FT‐ICR‐MS) and the duration DOM spent in anoxia (anaerobic duration, t_a_). A permutation test was run with 9999 permutations to determine significance of canonical analyses of t_a_^7,8^. The Spearman correlation coefficients ρ between the molecular dimensions (relative peak intensities from FT‐ICR‐MS) and the time axis were then calculated and indicate the strength and direction of the relationship. The ρ greater than 0.33 and less than − 0.33 were plotted in Van-Krevelen space. For this analysis, data across lakes was grouped because NMDS results did not show lakes as distinct populations of molecular composition. Additionally, we compared how individual formulae responded to anaerobic duration by binning formulae based on the formula nominal mass or NOSC. Bins were occupied with equal numbers of formulae to compare the value distribution of the Spearman correlation coefficient for each parameter and direction (formulae increasing and decreasing in anoxia, respectively)^46^. Significant differences among bins were explored using one-way analysis of variance (ANOVA) followed by Tukey's post hoc tests for multiple comparisons. All statistical analyses were performed with the *vegan* package in R (version 3.3.2).

### Supplementary Information

Supplementary Information 1.

Supplementary Information 2.

## Data Availability

The datasets used in the study are available in the supplementary material.

## References

[1] Cole JJ (2007). Plumbing the global carbon cycle: Integrating inland waters into the terrestrial carbon budget. Ecosystems.

[2] Tranvik LJ, Cole JJ, Prairie YT (2018). The study of carbon in inland waters-from isolated ecosystems to players in the global carbon cycle. Limnol. Oceanogr. Lett..

[3] Drake TW, Raymond PA, Spencer RGM (2018). Terrestrial carbon inputs to inland waters: A current synthesis of estimates and uncertainty. Limnol. Oceanogr. Lett..

[4] Kothawala DN (2014). Controls of dissolved organic matter quality: Evidence from a large-scale boreal lake survey. Glob. Chang. Biol..

[5] Williams CJ, Yamashita Y, Wilson HF, Jaffé R, Xenopoulos MA (2010). Unraveling the role of land use and microbial activity in shaping dissolved organic matter characteristics in stream ecosystems. Limnol. Oceanogr..

[6] Jaffé R (2008). Spatial and temporal variations in DOM composition in ecosystems: The importance of long-term monitoring of optical properties. J. Geophys. Res. Biogeosci..

[7] Hutchins RHS (2017). The optical, chemical, and molecular dissolved organic matter succession along a boreal soil-stream-river continuum. J. Geophys. Res. Biogeosci..

[8] Singer GA (2012). Biogeochemically diverse organic matter in Alpine glaciers and its downstream fate. Nat. Geosci..

[9] Zark M, Dittmar T (2018). Universal molecular structures in natural dissolved organic matter. Nat. Commun..

[10] Catalán N, Marcé R, Kothawala DN, Tranvik LJ (2016). Organic carbon decomposition rates controlled by water retention time across inland waters. Nat. Geosci..

[11] Vachon D, Prairie YT, Guillemette F, del Giorgio PA (2017). Modeling allochthonous dissolved organic carbon mineralization under variable hydrologic regimes in boreal lakes. Ecosystems.

[12] Kellerman AM, Dittmar T, Kothawala DN, Tranvik LJ (2014). Chemodiversity of dissolved organic matter in lakes driven by climate and hydrology. Nat. Commun..

[13] Hedges JI, Keil RG (1995). Sedimentary organic matter preservation: An assessment and speculative synthesis. Mar. Chem..

[14] Hemingway JD (2019). Mineral protection regulates long-term global preservation of natural organic carbon. Nature.

[15] Lalonde K, Mucci A, Ouellet A, Gélinas Y (2012). Preservation of organic matter in sediments promoted by iron. Nature.

[16] Hunter WR (2016). Metabolism of mineral-sorbed organic matter and microbial lifestyles in fluvial ecosystems. Geophys. Res. Lett..

[17] Tanentzap AJ (2019). Chemical and microbial diversity covary in fresh water to influence ecosystem functioning. Proc. Natl. Acad. Sci..

[18] Arrieta JM (2015). Dilution limits dissolved organic carbon utilization in the deep ocean. Science (80-).

[19] Hedges JI (1999). Sedimentary organic matter preservation; a test for selective degradation under oxic conditions. Am. J. Sci..

[20] Sobek S (2009). Organic carbon burial efficiency in lake sediments controlled by oxygen exposure time and sediment source. Limnol. Oceanogr..

[21] Bastviken D, Persson L, Odham G, Tranvik L (2004). Degradation of dissolved organic matter in oxic and anoxic lake water. Limnol. Oceanogr..

[22] Cole JJ, Pace ML (1995). Bacterial secondary production in oxic and anoxic freshwaters. Limnol. Oceanogr..

[23] Valle J (2018). Extensive processing of sediment pore water dissolved organic matter during anoxic incubation as observed by high-field mass spectrometry (FTICR-MS). Water Res..

[24] Boye K (2017). Thermodynamically controlled preservation of organic carbon in floodplains. Nat. Geosci..

[25] Brothers S (2014). A feedback loop links brownification and anoxia in a temperate, shallow lake. Limnol. Oceanogr..

[26] Peter S, Agstam O, Sobek S (2017). Widespread release of dissolved organic carbon from anoxic boreal lake sediments. Inl. Waters.

[27] Dadi T, Friese K, Wendt-Potthoff K, Koschorreck M (2016). Benthic dissolved organic carbon fluxes in a drinking water reservoir. Limnol. Oceanogr..

[28] Skoog AC, Arias-Esquivel VA (2009). The effect of induced anoxia and reoxygenation on benthic fluxes of organic carbon, phosphate, iron, and manganese. Sci. Total Environ..

[29] Knorr K-H (2013). DOC-dynamics in a small headwater catchment as driven by redox fluctuations and hydrological flow paths—Are DOC exports mediated by iron reduction/oxidation cycles?. Biogeosciences.

[30] Carey CC (2022). Anoxia decreases the magnitude of the carbon, nitrogen, and phosphorus sink in freshwaters. Glob. Chang. Biol..

[31] LaRowe DE, Van Cappellen P (2011). Degradation of natural organic matter: A thermodynamic analysis. Geochim. Cosmochim. Acta.

[32] Keiluweit M, Wanzek T, Kleber M, Nico P, Fendorf S (2017). Anaerobic microsites have an unaccounted role in soil carbon stabilization. Nat. Commun..

[33] Riedel T, Zak D, Biester H, Dittmar T (2013). Iron traps terrestrially derived dissolved organic matter at redox interfaces. Proc. Natl. Acad. Sci..

[34] Sowers TD, Holden KL, Coward EK, Sparks DL (2019). Dissolved organic matter sorption and molecular fractionation by naturally occurring bacteriogenic iron (oxyhydr)oxides. Environ. Sci. Technol..

[35] Lv J (2016). Molecular-scale investigation with ESI-FT-ICR-MS on fractionation of dissolved organic matter induced by adsorption on iron oxyhydroxides. Environ. Sci. Technol..

[36] Schiff SL (2017). Millions of boreal shield lakes can be used to probe archaean ocean biogeochemistry. Sci. Rep..

[37] Anderson NJ, Heathcote AJ, Engstrom DR (2020). Anthropogenic alteration of nutrient supply increases the global freshwater carbon sink. Sci. Adv..

[38] Feng M, Sexton JO, Channan S, Townshend JR (2016). A global, high-resolution (30-m) inland water body dataset for 2000: First results of a topographic–spectral classification algorithm. Int. J. Digit. Earth.

[39] Jane SF (2021). Widespread deoxygenation of temperate lakes. Nature.

[40] Gómez-Gener L, Lupon A, Laudon H, Sponseller RA (2020). Drought alters the biogeochemistry of boreal stream networks. Nat. Commun..

[41] Bartosiewicz M (2019). Hot tops, cold bottoms: Synergistic climate warming and shielding effects increase carbon burial in lakes. Limnol. Oceanogr. Lett..

[42] Maavara T (2020). River dam impacts on biogeochemical cycling. Nat. Rev. Earth Environ..

[43] Paulmier A, Ruiz-Pino D (2009). Oxygen minimum zones (OMZs) in the modern ocean. Prog. Oceanogr..

[44] LaBrie R, Hupfer M, Lau MP (2023). Anaerobic duration predicts biogeochemical consequences of oxygen depletion in lakes. Limnol. Oceanogr. Lett..

[45] Lapierre J-F, Guillemette F, Berggren M, del Giorgio PA (2013). Increases in terrestrially derived carbon stimulate organic carbon processing and CO_2_ emissions in boreal aquatic ecosystems. Nat. Commun..

[46] Drake TW (2019). Mobilization of aged and biolabile soil carbon by tropical deforestation. Nat. Geosci..

[47] Helms JR (2008). Absorption spectral slopes and slope ratios as indicators of molecular weight, source, and photobleaching of chromophoric dissolved organic matter. Limnol. Oceanogr..

[48] Koch BP, Dittmar T (2006). From mass to structure: An aromaticity index for high-resolution mass data of natural organic matter. Rapid Commun. Mass Spectrom..

[49] Hupfer M, Lewandowski J (2008). Oxygen controls the phosphorus release from lake sediments—A long-lasting paradigm in limnology. Int. Rev. Hydrobiol..

[50] Weyhenmeyer GA, Prairie YT, Tranvik LJ (2014). Browning of boreal freshwaters coupled to carbon–iron interactions along the aquatic continuum. PLoS One.

[51] Mendonça R (2017). Organic carbon burial in global lakes and reservoirs. Nat. Commun..

[52] Lau MP, Del Giorgio P (2020). Reactivity, fate and functional roles of dissolved organic matter in anoxic inland waters. Biol. Lett..

[53] Peter S, Isidorova A, Sobek S (2016). Enhanced carbon loss from anoxic lake sediment through diffusion of dissolved organic carbon. J. Geophys. Res. Biogeosci..

[54] Baines SB, Pace ML, Karl DM (1994). Why does the relationship between sinking flux and planktonic primary production differ between lakes and oceans?. Limnol. Oceanogr..

[55] Ferland M-E, Prairie YT, Teodoru C, del Giorgio PA (2014). Linking organic carbon sedimentation, burial efficiency, and long-term accumulation in boreal lakes. J. Geophys. Res. Biogeosciences.

[56] Mendonça R (2016). Organic carbon burial efficiency in a subtropical hydroelectric reservoir. Biogeosciences.

[57] Chmiel HE (2016). The role of sediments in the carbon budget of a small boreal lake. Limnol. Oceanogr..

[58] Vachon D, Sponseller RA, Karlsson J (2020). Integrating carbon emission, accumulation and transport in inland waters to understand their role in the global carbon cycle. Glob. Chang. Biol..

[59] Teodoru CR, Del Giorgio PA, Prairie YT, St-Pierre A (2013). Depositional fluxes and sources of particulate carbon and nitrogen in natural lakes and a young boreal reservoir in Northern Québec. Biogeochemistry.

[60] Hall BD (2019). Multidecadal carbon sequestration in a headwater boreal lake. Limnol. Oceanogr..

[61] Gudasz C (2017). Contributions of terrestrial organic carbon to northern lake sediments. Limnol. Oceanogr. Lett..

[62] Von Wachenfeldt E, Tranvik LJ (2008). Sedimentation in boreal lakes—The role of flocculation of allochthonous dissolved organic matter in the water column. Ecosystems.

[63] Shields MR, Bianchi TS, Gélinas Y, Allison MA, Twilley RR (2016). Enhanced terrestrial carbon preservation promoted by reactive iron in deltaic sediments. Geophys. Res. Lett..

[64] Björnerås C, Persson P, Weyhenmeyer GA, Hammarlund D, Kritzberg ES (2021). The lake as an iron sink-new insights on the role of iron speciation. Chem. Geol..

[65] Arndt S (2013). Quantifying the degradation of organic matter in marine sediments: A review and synthesis. Earth-Sci. Rev..

[66] Houser JN, Bade DL, Cole JJ, Pace ML (2003). The dual influences of dissolved organic carbon on hypolimnetic metabolism : Organic substrate and photosynthetic reduction. Biogeochemistry.

[67] Riedel T, Iden S, Geilich J, Wiedner K, Durner W, Biester H (2014). Changes in the molecular composition of organic matter leached from an agricultural topsoil following addition of biomass-derived black carbon (biochar). Org. Geochem..

[68] Stegen JC (2018). Influences of organic carbon speciation on hyporheic corridor biogeochemistry and microbial ecology. Nat. Commun..

[69] Bernhardt ES (2017). Control points in ecosystems: Moving beyond the hot spot hot moment concept. Ecosystems.

[70] Sleighter RL (2014). Evidence of incorporation of abiotic S and N into Prairie wetland dissolved organic matter. Environ. Sci. Technol. Lett..

[71] Wilson RM (2017). Hydrogenation of organic matter as a terminal electron sink sustains high CO_2_:CH_4_ production ratios during anaerobic decomposition. Org. Geochem..

[72] den Heyer C, Kalff J (1998). Organic matter mineralization rates in sediments: A within- and among-lake study. Limnol. Oceanogr..

[73] Carignan R, Planas D, Vis C (2000). Planktonic production and respiration in oligotrophic shield lakes. Limnol. Oceanogr..

[74] Vachon D, del Giorgio PA (2014). Whole-lake CO_2_ dynamics in response to storm events in two morphologically different lakes. Ecosystems.

[75] Reis PCJ, Thottathil SD, Prairie YT (2022). The role of methanotrophy in the microbial carbon metabolism of temperate lakes. Nat. Commun..

[76] Rasilo T, Prairie YT, del Giorgio PA (2015). Large-scale patterns in summer diffusive CH_4_ fluxes across boreal lakes, and contribution to diffusive C emissions. Glob. Chang. Biol..

[77] Cuthbert ID, Del Giorgio P (1992). Toward a standard method of measuring color in freshwater. Limnol. Oceanogr..

[78] Read, J. S. & Muraoka, K. *LakeAnalyzer*. Vol. 21 (2011).

[79] Quay PD, Broecker WS, Hesslein RH, Schindler DW (1980). Vertical diffusion rates determined by tritium tracer experiments in the thermocline and hypolimnion of two lakes1, 2. Limnol. Oceanogr..

[80] Livingstone DM, Imboden DM (1996). The prediction of hypolimnetic oxygen profiles: A plea for a deductive approach. Can. J. Fish. Aquat. Sci..

[81] Rippey B, McSorley C (2009). Oxygen depletion in lake hypolimnia. Limnol. Oceanogr..

[82] Dittmar T, Koch B, Hertkorn N, Kattner G (2008). A simple and efficient method for the solid-phase extraction of dissolved organic matter (SPE-DOM) from seawater. Limnol. Oceanogr. Methods.

[83] Hawkes JA (2020). An international laboratory comparison of dissolved organic matter composition by high resolution mass spectrometry: Are we getting the same answer?. Limnol. Oceanogr. Methods.

[84] Merder J (2020). ICBM-OCEAN: Processing ultrahigh-resolution mass spectrometry data of complex molecular mixtures. Anal. Chem..

