# Supplementary material for: The chemical succession in anoxic lake waters as source of molecular diversity of organic matter
Source: Sci Rep. 2024 Feb 15;14:3831. doi: 10.1038/s41598-024-54387-0 (PMC10869704; doi:10.1038/s41598-024-54387-0)
Supplement: Supplementary file 1 — Supplementary Information 1. [file 41598_2024_54387_MOESM1_ESM.zip › lau-et-al_data.pdf]

Data to submitted manuscript

The chemical succession in anoxic lake waters as source of molecular diversity of organic matter

Maximilian P Lau[1,2*], Ryan HS Hutchins[3,4], Suzanne E Tank[3], Paul del Giorgio[1]

1 Département des sciences biologiques, Université du Québec à Montréal (UQAM), 141 Avenue du Président-Kennedy, Montréal, Quebec, Canada H2X 1Y4

2 Interdisciplinary Environmental Research Centre, Technische Universität Bergakademie Freiberg, Brennhausgasse 14, D-09599 Freiberg, Germany

3 Department of Biological Sciences, University of Alberta, Edmonton, AB, T6G 2R3

4 Department of Chemistry and Biology, Toronto Metropolitan University, Toronto, ON M5B 2K3

**contact details of corresponding author: maximilian.lau@ioez.tu-freiberg.de*

1. Data Description

For a study of dissolved organic matter (DOM) composition and dynamic under anoxic conditions we survey two north-temperate lakes over the course of one year. We measured several physical and chemical properties of the water with in-situ sensors, loggers and in water samples. We conducted several experiments (assays) to study degradation kinetics under oxic and anoxic conditions and under light exposure. We used ultra-high-resolution mass spectrometry to analyze chemical composition of DOM.

1.1. Sampling method

Details on sampling methods can be found in the manuscript "The chemical succession in anoxic lake waters as source of molecular diversity of organic matter" by Lau et al. (submitted).

1.2. Analytical procedure:

Details on analytical procedures can be found in the manuscript "The chemical succession in anoxic lake waters as source of molecular diversity of organic matter" by Lau et al. (submitted).

1.3. Data processing

Details on data processing can be found in the manuscript "The chemical succession in anoxic lake waters as source of molecular diversity of organic matter" by Lau et al. (submitted).

2. File description

2.1. File inventory

Dataset contains the following data tables.

- 2023_scirep_lau-et-al_assays_photo
- 2023_scirep_lau-et-al_assays_bio
- 2023_scirep_lau-et-al_bathymetries
- 2023_scirep_lau-et-al_hrms
- 2023_scirep_lau-et-al_hrms-weighted-mean
- 2023_scirep_lau-et-al_observations_sondes_interpolated
- 2023_scirep_lau-et-al_observations_water_chemistry

2.2. Description of data tables

Files 2023_scirep_lau-et-al_assays_photo.csv and 2023_scirep_lau-et-al_assays_bio.csv

Column header	unit	Description
x		sample name and ID
campaign.no		sampling campaign (running number)
lake		sampling site (cc for Croche, cw for Cromwell)
mode		Mode of incubation assay (aerobic, anaerobic)
k	d^{-1}	first-order decay coefficient (rate)
lab.err, foto.err	same as rates	standard errors of decay coefficients
foto.decay.per.day	mg-C L^{-1} d^{-1}	linear rate of photochemical degradation in assays
anox.age	d	residence (time) in anoxic hypolimnetic water
diy	julian day	julian day

File 2023_scirep_lau-et-al_bathymetries.csv

Column header	unit	Description
depth.m	m	water depth
area	m^2	(surface) area of crosssection
lake		sampling site (cc for Croche, cw for Cromwell)

Files 2023_scirep_lau-et-al_hrms.csv and 2023_scirep_lau-et-al_hrms-weighted-mean.csv (for intensity-normalized means of several properties).

Column header	unit	Description
x		sample name and ID
mz	m z^{-1}	mass-to-charge ratio
formula		molecular formula
H, O, N, S, P	n	elements in formula
ResPow		Resolving power
O:C		Elemental ratios (mol mol-1)
AI.mod		Aromaticity index
DBE	n	Double bond units
NOSC		Nominal oxidation state of Carbon
V1		Spearman‘s rank correlation coefficient

File 2023_scirep_lau-et-al_observations_sondes_interpolated.csv

Column header	unit	Description
depth	m	water depth
t.c	°C	temperature
o2.mgL	mg L^{-1}	dissolved oxygen concentration
cond	µS cm^{-1}	electrical conductivity
pH		pH
lake		sampling site (cc for Croche, cw for Cromwell)
campaign.no		sampling campaign (running number)

Values „100“ indicate NA

File 2023_scirep_lau-et-al_observations_water_chemistry.csv

Column header	unit	Description
id2	-	sample name and ID
campaign.no	-	sampling campaign (running number)
depth.x	m	water depth
doc, dic	mg-C L^{-1}	dissolved organic and inorganic carbon
ch4.umol	µmol L^{-1}	dissolved methane
spec.slo	-	Spectral Slope Ratio according to Helms et al. 2008
anox.age	d	residence (time) in anoxic hypolimnetic water
stag.age, mix.age	d	residence (time) in oxic hypolimnetic water
fe.ugL	µg L^{-1}	dissolved iron (Fe) concentration
cdom	m^{-1}	chromophoric dissolved organic carbon cDOM
