# Supplementary material for: The chemical succession in anoxic lake waters as source of molecular diversity of organic matter
Source: Sci Rep. 2024 Feb 15;14:3831. doi: 10.1038/s41598-024-54387-0 (PMC10869704; doi:10.1038/s41598-024-54387-0)
Supplement: Supplementary file 2 — Supplementary Information 2. [file 41598_2024_54387_MOESM2_ESM.pdf]

Manuscript submitted to Scientific Reports
Supplementary Information

Title

The chemical succession in anoxic lake waters as source of molecular diversity of organic matter

Authors

Maximilian P Lau[1,2*], Ryan HS Hutchins[3,4], Suzanne E. Tank[3], Paul A. del Giorgio[2]

1 Interdisciplinary Environmental Research Centre, Technische Universität Bergakademie Freiberg, Brennhausgasse 14, D-09599 Freiberg, Germany
2 Département des sciences biologiques, Université du Québec à Montréal (UQAM), 141 Avenue du Président-Kennedy, Montréal, Quebec, Canada H2X 1Y4
3 Department of Biological Sciences, University of Alberta, Edmonton, AB, T6G 2R3
4 Department of Chemistry and Biology, Toronto Metropolitan University, Toronto, ON M5B 2K3

*contact details of corresponding author: maximilian.lau@ioez.tu-freiberg.de

Keywords
lake-water quality, anoxia, carbon cycling, chemical diversity

Supplemental Figures S1-S3

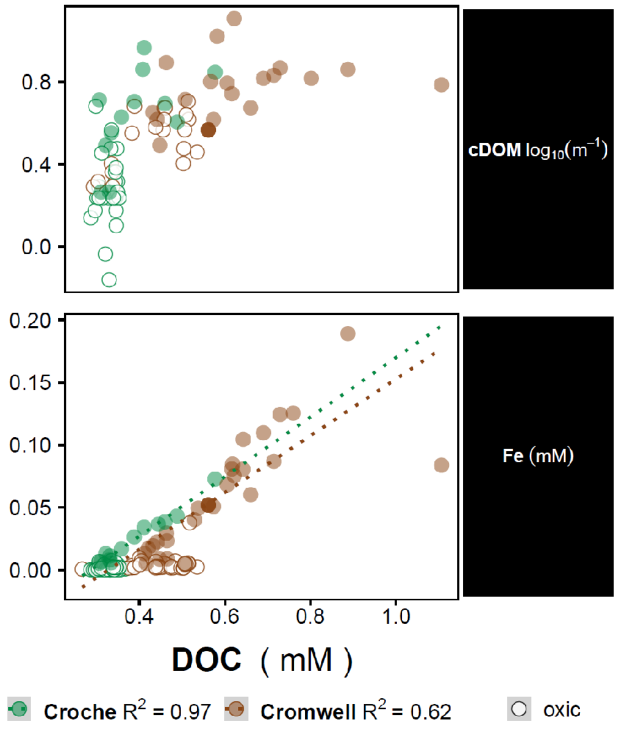

Figure S1 (a) Colored DOM (cDOM, absorption at 440nm) and **(b)** concentrations of Fe related to DOC concentration in waters of the two north temperate lakes. Filled dots indicate samples from the anoxic water column. Empty dots show samples taken from the well-mixed epilimnetic waters. Lines show linear models to the anoxic data.

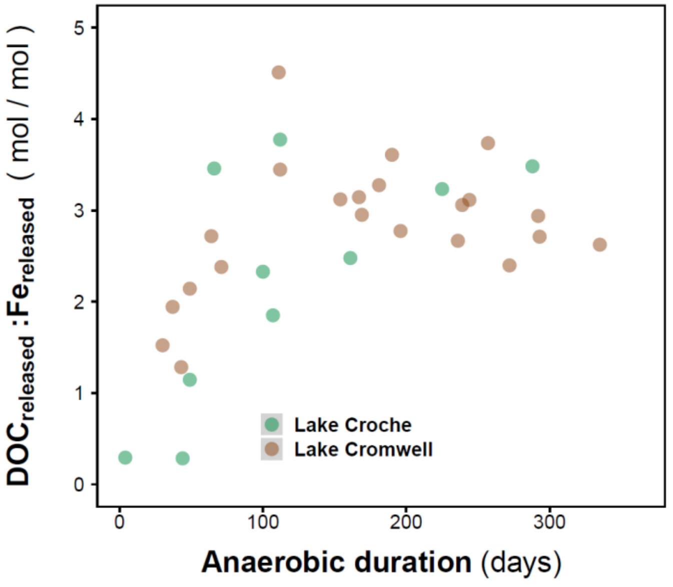

Figure S2: Molar C:Fe ratios of additional DOC in the anoxic water columns throughout different anaerobic duration (t_a, uninterrupted anoxic conditions). Ratios for the newly added DOM were calculated by subtraction of Fe and DOC concentrations at the onset of anoxia.

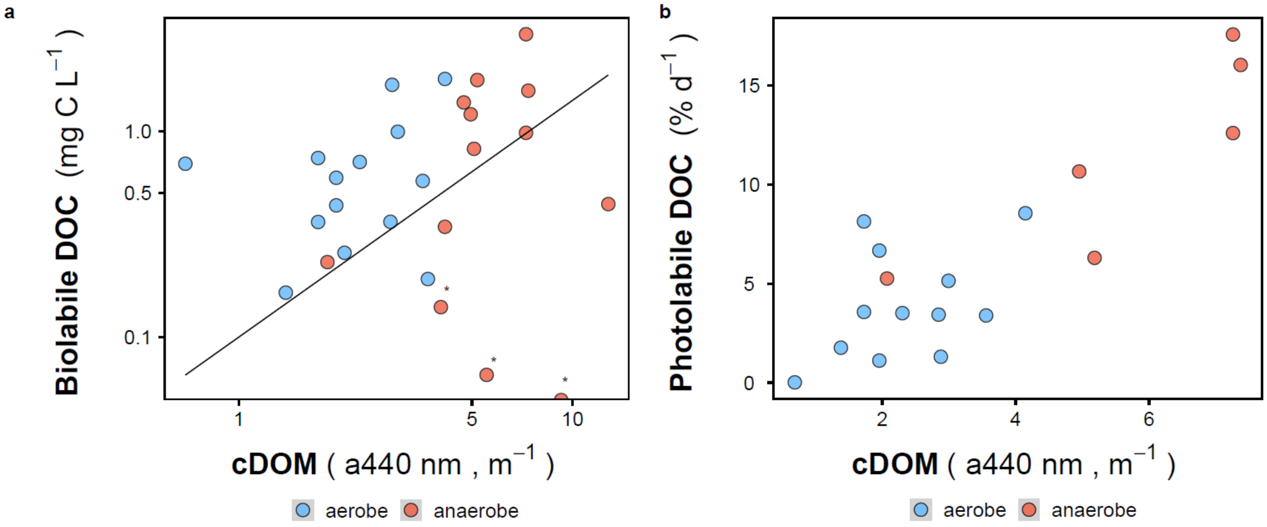

Figure S3: (a) Biolabile DOC from bottle incubation experiments expressed in absolute amounts of C mineralized after 14 days of incubation in relation to DOC color (absorption at 440nm). Previously, DOM color was found to increase with its biolabile fraction (solid line depicts the empirical model from Lapierre et al.[45]). In comparison to their color, three samples returned unrealistically low results in their biolability assays (asterisk). In comparison to their color, three samples returned unrealistically low results in their biolability assays (asterisks). **(b)** Photolabile DOC in relation to DOC color.

Impact of DOC release on sediment carbon budget

First, volumetric anaerobic duration distributions were computed for each lake. Each pair of date and depth holds water of a particular t_a. We accumulated the volumes of the date-depth increments (1- layer, 1 day) of similar t_a (t_{a1}, t_{a2}, …) from all days in a full mixis-mixis period. The assembled anaerobic duration distribution, $V(t_a)$, then represents the anoxia-affected volumes for all values of t_a. Anoxic conditions prevailed in 4.6% (Croche) and 9.2% (Cromwell) of the lakes' total spatiotemporal extend (365 days ▪ $volume_{tot}$) between two full mixing events in 2017 and 2018. DOC accumulation rates (in mg_{DOC} L^{-1} d^{-1}) are expressed here as averages for the anoxic hypolimnion. Through normalization by the lake size, areal rates can be computed. Formula S1 shows the calculation for Lake Cromwell

$$DOC\ release = 1.2\ \frac{g_{DOC}}{m^2\ yr} = \frac{0.010\,\frac{mg_{DOC}}{L\,d}\,(365\,\frac{d}{yr}\cdot 359'000\ m^3\cdot 9.2\%)}{102'000\ m^2} \qquad \text{(Formula S1)}$$

For Cromwell, we assume an OCBE of 41% and a total sedimentation flux between 10 and 50 g_C m^{-2} yr^{-1} (Ferland et al. [55], Formula 6). During early diagenesis, 4-21% of this flux may be actually released as DOC (instead of CO_2).

Biolability increase in anoxic waters

We explored the relationship between total DOC and biolabile DOC in samples from the anoxic hypolimnion. Slopes of linear regressions to the data in fugure 3c were significantly positively increasing in case of lake Cromwell, but not in lake Croche. The comparison of biolability with cDOM and other studies showed that 3 samples had unrealistically low biolability values (i.e., degradation kinetics in bioassay), indicated in Figure S3a with asterisk symbols. If these values are considered outliers, and excluded from the analysis, the regression to the data in figure 3c are also significant for lake Croche (Table S1, grey).

Table S1: Linear regression coefficients and statistics of data in figure 3c

**Lake**	**Slope**	***R^2***	**p**
Cromwell	0.76<br>0.72 [a]	0.91<br>0.94 [a]	**<0.01**<br>**<0.01** [a]
Croche	0.22<br>0.7 [a]	0.06<br>0.90 [a]	0.6<br>**0.01** [a]

[a] After removal of potential outliers (values with asterisk in Figure S3a)
